# Endometriosis Is Associated with an Increased Risk of Coronary Artery Disease in Asian Women

**DOI:** 10.3390/jcm10184173

**Published:** 2021-09-15

**Authors:** Pei-Chen Li, Yu-Cih Yang, Jen-Hung Wang, Shinn-Zong Lin, Dah-Ching Ding

**Affiliations:** 1 Department of Obstetrics and Gynecology, Hualien Tzu Chi Hospital, Buddhist Tzu Chi Medical Foundation, Tzu Chi University, Hualien 970, Taiwan; ppchen24@gmail.com; 2 Management Office for Health Data, China Medical University Hospital, Taichung 404, Taiwan; moiluvkiwi@gmail.com; 3 College of Medicine, China Medical University, Taichung 404, Taiwan; 4 Department of Research, Hualien Tzu Chi Hospital, Buddhist Tzu Chi Medical Foundation, Tzu Chi University, Hualien 970, Taiwan; jenhungwang2011@gmail.com; 5 Department of Neurosurgery, Hualien Tzu Chi Hospital, Buddhist Tzu Chi Medical Foundation, Tzu Chi University, Hualien 970, Taiwan; shinnzong@yahoo.com.tw; 6 Institute of Medical Sciences, Tzu Chi University, Hualien 970, Taiwan

**Keywords:** coronary artery disease, endometriosis, hypertension, hyperlipidemias, diabetes mellitus

## Abstract

Endometriosis is a common systemic chronic inflammatory disease. Inflammation is the key mechanism responsible for the development of endothelial dysfunction and atherosclerosis. We aimed to investigate the risk of coronary artery disease (CAD) among Asian women with endometriosis. This retrospective population-based cohort study included patients with endometriosis diagnosed from 2000 to 2012 and registered in the Longitudinal Health Insurance Database, Taiwan. The comparison cohort (those without endometriosis) were selected (1:4) by matching the age frequency and the index year. We followed up the patients until the diagnosis of CAD (ICD-9-CM codes: 410–414, A270, and A279), withdrawal from the National Health Insurance system, death, or the end of the study. We used a multivariable-adjusted Cox proportional hazard model for evaluating the risk of CAD. We included 19,454 patients with endometriosis and 77,816 women as a comparison group. The mean age of the women at the diagnosis of endometriosis was 37.4 years. A total of 3245 women developed CAD in both groups during a median follow-up of 7 years. The incidence of CAD was higher in women with endometriosis than in those without (5.96 vs. 4.38 per 10,000 person-years; adjusted hazard ratio [95% confidence interval], 1.34 [1.22–1.47]). In conclusion, Asian women with endometriosis had a significantly higher risk of CAD. Further large-scale studies are needed to elucidate the cause-effect relationship between endometriosis and CAD.

## 1. Introduction

Endometriosis is a systemic chronic inflammatory disease that affects 5–10% of the women of reproductive age in the United States [1]. The prevalence of endometriosis is higher in Asian women (approximately 6.8–15.7%) than in Caucasian women [2,3]. Endometriosis is characterized by the presence of endometrial tissues outside the uterus, leading to dysmenorrhea, dyspareunia, pelvic or abdominal pain, and infertility. The exact origin and pathogenesis of endometriosis remain unclear. The American Society for Reproductive Medicine (ASRM) criteria is mostly used to define the severity of endometriosis [4]. Based on endometriosis lesion’s location and size, ASRM criteria classify endometriosis from stage 1 (minimal disease, point score 1–5) to stage IV (severe disease, point score >40) [4].

Emerging evidence indicates that endometriosis is linked to several chronic diseases, including atherosclerosis, coronary heart disease, dyslipidemia, hypertension, autoimmune disease, and even gynecologic cancers [5,6,7,8,9,10,11]. Several inflammatory cytokines, such as the intercellular adhesion molecule 1, C-reactive protein, interleukins 1 and 6, tumor necrosis factor-α, and vascular endothelial growth factor, are elevated in both the serum and the peritoneal fluid of women with endometriosis, suggesting that endometriosis is associated with local and systemic inflammation [6,12]. Recent evidence indicates higher oxidative stress and an atherogenic lipid profile in women with endometriosis, indicating a mechanism similar to that of endothelial dysfunction and atherosclerosis [11,13,14].

Coronary artery disease (CAD) is the leading cause of morbidity and mortality for all women, particularly postmenopausal women [15]. However, recent studies have shown an increased prevalence of CAD in young women (<55 years), with no improvements in the prevalence and mortality rates of CAD in this age group over the last few decades [16,17]. It is imperative that we examine the varying risk factors of CAD in young women [18]. Women with endometriosis may represent a high-risk population for the development of CAD due to chronic inflammation and atherosclerosis [7].

Hence, we investigated the risk of CAD among Asian women with endometriosis.

## 2. Materials and Methods

### 2.1. Ethics

This study was approved by the Institutional Review Board of China Medical University and the Hospital Research Ethics Committee (IRB permit number: CMUH-104-REC2-115) and is in compliance with institutional guidelines. Written informed consent from patients was waived due to low risk, and the study was approved by the institutional IRB of China Medical University and the Hospital Research Ethics Committee.

### 2.2. Data Source

This was a retrospective population-based cohort study. The data were retrieved from the Taiwan National Health Insurance Research Database (NHIRD). The purpose of creating the NHIRD was to generate reference data for medical and health policies and enhance medical research resources in Taiwan. The NHIRD includes numerous registration records of the general population in Taiwan, including clinical visits, hospitalizations, diagnoses, prescriptions, medical costs for reimbursement, and residential areas. All patient-related information was encrypted for confidentiality. The diagnoses in the NHIRD were coded by physicians in accordance with the International Classification of Disease, 9th Revision, Clinical Modification (ICD-9-CM) between 2000 and 2013.

### 2.3. Study Participants

We selected 19,454 patients newly diagnosed with endometriosis, which were incident cases, (ICD-9-CM code: 617) between 2000 and 2012 from the Longitudinal Health Insurance. The comparison group (those without endometriosis; N = 77,816) were randomly selected from the NHIRD by matching the age frequency and the index year. Patients above 64 years old or diagnosed with CAD before the index date were eliminated. We followed up with the patients until the diagnosis of CAD, withdrawal from the National Health Insurance system, death, or the end of the study (31 December 2013).

### 2.4. Outcomes and Comorbidities

The outcome of interest was the incidence of CAD (ICD-9-CM codes: 410–414, A270, and A279) after the index date. The following conditions that are known to be associated with CAD were evaluated as a potential confounder including obesity (ICD-9-CM codes: 278 and A183), and risk factors of CAD including chronic kidney disease (ICD-9-CM codes: 585 and 586), hypertension (ICD-9-CM codes: 401–405, A260, and A269), hyperlipidemia (ICD-9-CM codes: 272.0–272.4), and diabetes mellitus (ICD-9-CM code: 250) [19,20]. We also considered the role of surgeries (including hysterectomy and oophorectomy) and medication (including hormone, statin, aspirin, antihypertensive therapy, diabetes medication, or insulin therapy) on the risk of CAD [21].

### 2.5. Statistical Analyses

Categorical variables were compared using univariate analysis with the chi-square test. Continuous variables were compared by the Student’s t-test. We calculated person-years as the sum of the follow-up period for each individual. The incidence rate was calculated by the number of events and person-years. Univariate and multivariable estimated–hazard ratios (HRs) and 95% confidence intervals (CI) were calculated by the Cox proportional hazard model. Besides, we looked into the interaction between the potential confounders and endometriosis by including an interaction term into the multivariable model. The cumulative incidence of endometriosis to CAD was assessed by Kaplan–Meier analysis and the log-rank test. A two-tailed *p*-value of <0.05 and a 95% CI not including 1 were deemed statistically significant. All data were analyzed by SAS version 9.4 (SAS Institute Inc., Cary, NC, USA) for Windows 10.

## 3. Results

### 3.1. Subject Characteristics

We included 19,454 women newly diagnosed with endometriosis and 77,816 women without endometriosis. The mean age during the diagnosis of endometriosis was 37.4 ± 8.95 years. The mean follow-up duration was 7.36 (±3.82) years in the endometriosis cohort and 7.02 (±3.86) years in the comparison cohort (Table 1). There were no significant differences between the endometriosis cohort and the comparison cohort in terms of the age at entry in the cohort and the mean follow-up duration—meaning, the frequency matching was successful. However, the proportion of women with obesity, hypertension, hyperlipidemia, DM, past history of hysterectomy, oophorectomy, hormonal treatment, statin, aspirin, antihypertensive, and antihyperglycemic drug were significantly higher in the endometriosis cohort than in the comparison cohort.

### 3.2. Risk of CAD

**Figure 1:**
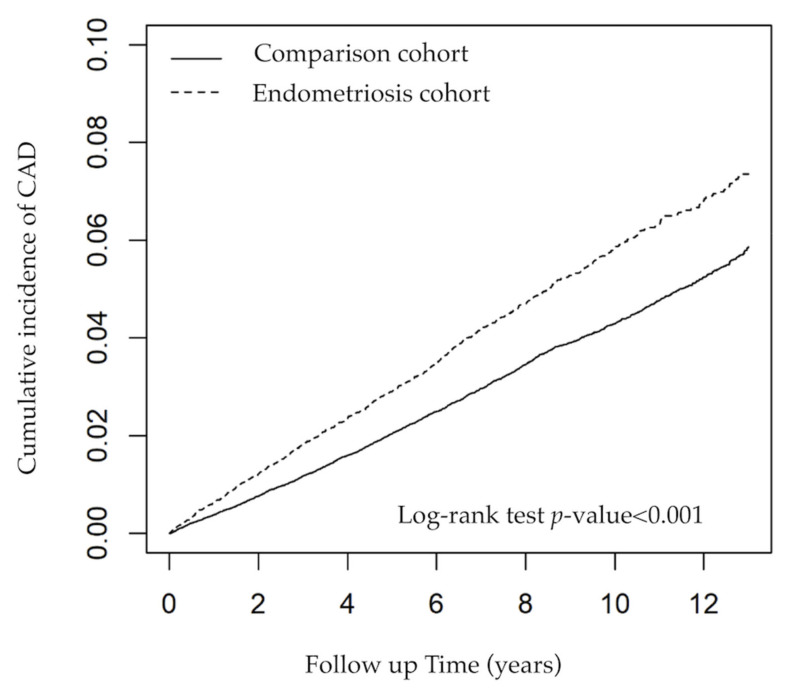
Kaplan–Meier analysis with unadjusted cumulative incidence curves of CAD for endometriosis cohort and comparison cohort.

Table 2 shows the incidence rates and adjusted HRs of CAD. A total of 3245 patients developed CAD. The incidence of CAD was higher in the endometriosis cohort than in the comparison cohort (5.96 vs. 4.38 per 1000 person-years; adjusted HR [95% CI], 1.34 [1.22, 1.47]). Figure 1 shows that the cumulative incidence of CAD events was significantly higher in the endometriosis cohort than in the comparison cohort (log-rank test, *p* < 0.001).

### 3.3. Subgroup Analysis According to Age

Endometriosis was associated with CAD in age groups <40 and 40–49. The adjusted HRs (95% CI) for endometriosis patients were 1.42 (1.19, 1.70) and 1.33 (1.18, 1.49) in age groups <40 and 40–49, respectively, relative to patients without endometriosis (Table 3). The association was not significant in the age group 50–64.

### 3.4. Subgroup Analysis According to Comorbidities and Medicine

In patients with hypertension, endometriosis increased the risk of CAD by 1.24-fold (1.04, 1.48) compared to the comparisons. Similarly, patients with endometriosis who took antihypertensive drugs had a higher hazard ratio of CAD, (adjusted HR = 1.12; 95% CI = 1.01, 1.24), see Table 3. Moreover, there were interactions between endometriosis and age, hypertension, hyperlipidemia, DM, oophorectomy, statin, and antihyperglycemic drugs.

Taken together, age and hypertension were associated with the risk of CAD in women with endometriosis in comparison with women without endometriosis (Table 3).

### 3.5. Subgroup Analysis According to Comorbidities and Medicine in Endometriosis Patients

In endometriosis patients, increasing age was associated with an increased risk of CAD (Table 4). Obesity, hypertension, hyperlipidemia, and DM were also associated with an increased risk of CAD (Table 4). Regarding medication, hormone, statin, and anti-hyperglycemic medication were associated with a decreased risk of CAD (Table 4). However, aspirin and anti-hypertensive medication were associated with an increased risk of CAD in endometriosis patients (Table 4).

## 4. Discussion

In this study, we found that women of age groups below 50 years with endometriosis had a higher risk of CAD as compared to those without. After 7.37 years (median) of follow-up, women with endometriosis had a 1.27-fold overall risk of developing CAD. Interestingly, we discovered a prominent unpropitious effect in patients without chronic kidney disease, hypertension, hyperlipidemia, diabetes mellitus, hysterectomy, oophorectomy, and medical treatment. Our findings suggest that healthy individuals, even without the known risk factors of CAD, should be aware of the risk of CAD when diagnosed with endometriosis.

In addition to endometriosis, the risk of CAD was associated with age, obesity, chronic kidney disease, hypertension, hyperlipidemia, and diabetes mellitus, which are well-known risk factors of CAD. CAD begins with atherosclerosis, which is an inflammatory state of the intima of large and medium-sized arteries [22]; inflammation also leads to local, myocardial, and systemic complications of atherosclerosis. The above risk factors could further exaggerate the progression of atherosclerosis. In our study, we also found obesity, hypertension, hyperlipidemia, and DM were also associated with an increased risk of CAD in endometriosis patients.

A comparison of women with and without endometriosis revealed that the adjusted HRs for CAD were the highest among those younger than 40 years of age and the risk decreased with age. These results were consistent with those of a previous study [7]. CAD is an age-related disease. Previous studies have shown that cytokine dysregulation results in the loss of the regulation of systemic inflammation at an older age [23]. Contrary to endometriosis, increasing age may be more strongly associated with CAD in elderly women. In our study, we also found increasing age was also associated with an increased risk of CAD in endometriosis patients (HR: 50–65 years > 40–49 years > below 40 years).

The association between endometriosis and cardiovascular disease depicts an emerging topic in the field of women’s health. Various studies have emphasized immune mediators in endometriosis causing chronic inflammation, suggesting that endometriosis is a systemic, rather than a localized condition [24,25,26,27]. Endometriosis has currently been redefined as a systemic inflammatory disease. Endometriosis and atherosclerosis share similar mechanisms involving increased activated macrophages and inflammatory cytokines [28]. Women with endometriosis were found to have more macrophages, neutrophils, and dendritic cells in the peritoneal fluid than those without [29]. A recent study by Weisheng et al. detected 260 cytokines, chemokines, and growth factors in serum samples of patients with endometriosis [30]. Women with endometriosis also have significantly higher levels of serum markers of endothelial inflammation and activation, including the vascular cell adhesion molecule-1, intercellular adhesion molecule-1, E-selectin, vascular endothelial growth factor, von Willebrand factor, and ristocetin cofactor, reflecting an early development of atherosclerosis [31]. In several studies assessing ultrasonographic parameters such as a common carotid intima-media thickness, distensibility coefficient, flow-mediated dilation, and pulse wave velocity, women with endometriosis have shown increased arterial stiffness than the general population [32,33]. Studies have also suggested that oxidative stress and the presence of oxidized low-density lipoprotein in the blood might contribute to both diseases [5].

Aspirin (ASA) can be used for the primary and secondary prevention of CAD [34]. In previous clinical trials, the risk of CAD after taking ASA could be reduced by 20–40% [35,36,37,38]. However, one study showed low dose ASA did not have the protective effect of CAD [39]. Statin can also be used for the primary prevention of CAD [40]. The previous meta-analysis analyzed the 16 randomized control trials and showed statin appeared to be favorable for primary prevention of CAD but without effect on CAD death [40]. In our study, we found statin was also associated with a decreased risk of CAD in endometriosis patients. However, ASA was associated with an increased risk of CAD in endometriosis patients. We speculate the dosage of ASA in our population may not be enough for the prevention of CAD. In Taiwan, the usual dosage of ASA is 100 mg per day which may be considered a low dose.

Oral contraceptives contain estrogen and progestin may be used in the treatment of endometriosis, which can reduce the risk of endometriosis [41]. Our study showed 58% of endometriosis patients used hormone medication. Hormone use also decreases CAD risk in endometriosis patients in our study. DM is a risk factor and comorbidity of CAD. In DM patients, antiglycemic medication will affect the outcome of CAD [42]. In our study, antiglycemic medications decreased the risk of CAD in endometriosis patients.

Our study adds evidence on the association between endometriosis and adverse cardiovascular events. Prospective studies by Mu et al. involving 116,430 women from the Nurses’ Health Study II cohort reported that endometriosis was associated with an increased risk of coronary heart disease (relative risk [RR], 1.62; 95% CI, 1.39–1.89), hypercholesterolemia (RR, 1.25; 95% CI, 1.21–1.30), and hypertension (RR, 1.14; 95% CI, 1.09–1.18) [7,8]. A population-based cohort study by Chiang et al. demonstrated that women with endometriosis had a 1.2-fold increased risk of major adverse cardiovascular and cerebrovascular events (95% CI 1.05–1.29; *p* = 0.0053) as compared to the general population [43].

Our study should raise awareness on cardiovascular health in women by highlighting the role of inflammation in patients with endometriosis. Early diagnosis and treatment of endometriosis may prevent subsequent cardiovascular complications and warrant a better quality of life in these patients.

Our study has several strengths. We included a large population-based cohort to investigate the association between endometriosis and CAD. We adjusted for several comorbidities, such as obesity, chronic kidney disease, hypertension, hyperlipidemia, diabetes mellitus, history of hysterectomy, oophorectomy, and hormonal treatment, which are the known risk factors of CAD. Our patients were representative of the general population, and the comparison cohort was enrolled from the same population.

However, there are several limitations to this study. First, the gold standard of diagnosis of endometriosis is laparoscopic visualization with or without histological confirmation. In our study, endometriosis was diagnosed by invasive or non-invasive diagnostic methods based on the ICD-9-CM codes [38]. This may impact the results likely driving the association observed toward or away from the null. Invasive methods such as laparoscopic tissue proof were the definite diagnosis for endometriosis. Non-invasive methods such as ultrasound and tumor markers may diagnose endometriosis as well. However, non-invasive diagnosis methods are non-specific and may contain women without endometriosis, which may increase the association. Second, personal and social history, such as cigarette smoking, physical activity, and family history, could not be obtained from the NHIRD. Third, the confounders for endometriosis such as parity and age at menarche were not recorded in the database. Increased parity may be associated with a decreased risk of endometriosis. A previous study showed parity ≥2 had an odds ratio 0.3 (95% CI: 0.1–0.6) [44]. However, women with one child were not associated with a decreased risk of endometriosis (OR: 0.7, 95% CI: 0.3–1.7) [44]. Another study also reported parity was inversely associated with the risk of endometriosis [45]. Early menarche (less than 11–12 years of age) was reported associated with the risk of endometriosis [46].

In conclusion, endometriosis was significantly associated with a higher risk of CAD in Asian women. Further large-scale studies are needed to elucidate the cause-effect relationship between endometriosis and CAD.

## Figures and Tables

**Table 1:** Baseline characteristics of the women in the endometriosis and comparison cohorts.

	Endometriosis	Comparison Group	
	(*n* = 19,454)	(*n* = 77,816)	*p*-Value
Age, Years, *n* (%)			>0.999
<40	11,049 (56.80)	44,196 (56.80)	
40–49	7268 (37.36)	29,072 (37.36)	
50–64	1137 (5.84)	4548 (5.84)	
Mean (SD)	37.37 (8.95)	37.34 (9.13)	0.658
Comorbidity, *n* (%)			
Obesity	232 (1.19)	641 (0.82)	<0.001
CKD	68 (0.35)	263 (0.34)	0.804
Hypertension	1472 (7.57)	4894 (6.29)	<0.001
Hyperlipidemia	1269 (6.52)	3862 (4.96)	<0.001
DM	965 (4.96)	3003 (3.86)	<0.001
Hysterectomy	4709 (24.21)	1769 (2.27)	<0.001
Oophorectomy	4861 (24.99)	1802 (2.32)	<0.001
Medication, *n* (%)			
Hormone (estradiol, premarin)	11,331 (58.25)	28,362 (36.45)	<0.001
Statin	1886 (9.69)	5728 (7.36)	<0.001
Aspirin	9074 (46.64)	29,210 (37.54)	<0.001
Antihypertensive	11,639 (59.83)	35,399 (45.49)	<0.001
Antihyperglycemic	1441 (7.41)	4565 (5.87)	<0.001
Insulin	399 (2.05)	1401 (1.80)	0.020
Follow-up duration, years			
Mean (SD)	7.36 (3.82)	7.02 (3.86)	<0.001

CKD: coronary kidney disease; DM: diabetes mellitus.

**Table 2:** Cox model-measured hazard ratios and 95% confidence intervals of CAD associated with gender, age, and comorbidity per 1000 person-years.

Endometriosis	N	CAD Event	Person-Years	IR	HR (95% CI)	
					Crude	Adjusted ^†^
No	77,816	2392	546,412	4.38	1.00 (reference)	1.00 (reference)
Yes	19,454	853	143,169	5.96	1.36 (1.26, 1.47) ***	1.34 (1.22, 1.47) ***

N: number of patients; CAD: coronary artery disease; IR: incidence rates per 1000 person-years; HR: hazard ratio; CI: confidence interval; ^†^ Model was adjusted for age, comorbidities, and medication listed in Table 1. *** *p* < 0.001.

**Table 3:** Comparison of the incidence rates and hazard ratios of CAD between the two groups stratified by age, comorbidities, and medication.

	Endometriosis Cohort			Comparison Cohort			Crude		Adjusted ^†^		
	CAD Event	PY	IR	CAD Event	PY	IR	HR (95% CI)	*p*-Value	HR (95% CI)	*p*-Value	*p*-Value for Interaction
Age, years											<0.001
<40	230	84,798	2.71	529	314,233	1.68	1.60 (1.37, 1.86)	<0.001	1.42 (1.19, 1.70	<0.001	
40–49	505	51,333	9.84	1425	204,036	6.98	1.41 (1.27, 1.56)	<0.001	1.33 (1.18, 1.49)	<0.001	
50–64	118	7038	16.77	438	28,143	15.56	1.08 (0.88, 1.32)	0.475	1.02 (0.81, 1.29)	0.870	
Comorbidity											
Obesity											0.438
No	831	141,770	5.86	2358	542,807	4.34	1.35 (1.24, 1.46)	<0.001	1.17 (1.07, 1.28)	<0.001	
Yes	22	1399	15.73	34	3605	9.43	1.66 (0.97, 2.84)	0.064	1.42 (0.76, 2.65)	0.270	
CKD											0.096
No	844	142,741	5.91	2351	544,958	4.31	1.37 (1.26, 1.48)	<0.001	1.18 (1.08, 1.30)	<0.001	
Yes	9	428	21.03	41	1454	28.21	0.76 (0.37, 1.56)	0.454	0.80 (0.35, 1.87)	0.611	
Hypertension											0.047
No	632	133,732	4.73	1753	514,840	3.40	1.38 (1.26, 1.51)	<0.001	1.16 (1.04, 1.29)	0.007	
Yes	221	9437	23.42	639	31,572	20.24	1.16 (0.99, 1.35)	0.062	1.24 (1.04, 1.48)	0.017	
Hyperlipidemia											<0.001
No	735	135,336	5.43	2032	523,695	3.88	1.40 (1.28, 1.52)	<0.001	1.22 (1.11, 1.35)	<0.001	
Yes	118	7833	15.06	360	22,716	15.85	0.96 (0.78, 1.18)	0.690	0.96 (0.76, 1.22)	0.747	
DM											0.030
No	734	136,463	5.38	2065	526,736	3.92	1.37 (1.26, 1.49)	<0.001	1.18 (1.07, 1.30)	0.001	
Yes	119	6706	17.74	327	19,676	16.62	1.07 (0.87, 1.32)	0.523	1.16 (0.91, 1.48)	0.222	
Hysterectomy											0.267
No	540	105,475	5.12	2281	532,435	4.28	1.19 (1.09, 1.31)	<0.001	1.23 (1.11, 1.36)	<0.001	
Yes	313	37,694	8.30	111	13,977	7.94	1.04 (0.84, 1.30)	0.690	1.09 (0.88, 1.35)	0.443	
Oophorectomy											0.004
No	662	104,720	6.32	2323	533,103	4.36	1.45 (1.33, 1.58)	<0.001	1.22 (1.11, 1.35)	<0.001	
Yes	191	38,449	4.97	69	13,309	5.18	0.95 (0.72, 1.25)	0.724	1.05 (0.79, 1.39)	0.735	
Medication											
Hormone (estradiol + premarin)											0.089
No	342	56,806	6.02	1424	340,439	4.18	1.44 (1.28, 1.62)	<0.001	1.29 (1.12, 1.47)	<0.001	
Yes	511	86,363	5.92	968	205,972	4.70	1.25 (1.12, 1.39)	<0.001	1.11 (0.98, 1.25)	0.103	
Statin											0.001
No	704	127,306	5.53	1948	498,615	3.91	1.42 (1.30, 1.54)	<0.001	1.20 (1.09, 1.33)	<0.001	
Yes	149	15,863	9.39	444	47,797	9.29	1.01 (0.84, 1.22)	0.917	1.08 (0.87, 1.35)	0.494	
Aspirin											0.004
No	379	75,191	5.04	1145	336,187	3.41	1.48 (1.32, 1.66)	<0.001	1.27 (1.11, 1.46)	<0.001	
Yes	474	67,978	6.97	1247	210,225	5.93	1.17 (1.06, 1.30)	0.003	1.11 (0.98, 1.25)	0.105	
Antihypertensive											0.008
No	157	54,941	2.86	548	282,990	1.94	1.48 (1.24, 1.76)	<0.001	1.46 (1.20, 1.79)	<0.001	
Yes	696	88,228	7.89	1844	263,422	7.00	1.13 (1.03, 1.23)	0.008	1.12 (1.01, 1.24)	0.029	
Antihyperglycemic											<0.001
No	755	131,763	5.73	2043	510,470	4.00	1.43 (1.32, 1.55)	<0.001	1.22 (1.11, 1.34)	<0.001	
Yes	98	11,406	8.59	349	35,942	9.71	0.88 (0.71, 1.11)	0.285	0.94 (0.72, 1.22)	0.652	
Insulin											0.152
No	813	140,141	5.80	2262	536,065	4.22	1.37 (1.27, 1.49)	<0.001	1.18 (1.08, 1.30)	<0.001	
Yes	40	3028	13.21	130	10,347	12.56	1.06 (0.74, 1.51)	0.764	1.16 (0.76, 1.79)	0.491	

IR: incidence rates per 1000 person-years; HR: hazard ratio; CI: confidence interval; †: Model was adjusted for age and comorbidities listed in Table 1. CAD: coronary artery disease; CKD: coronary kidney disease; DM: diabetes mellitus.

**Table 4:** Comparison of the incidence rates and hazard ratios of CAD in endometriosis patients stratified by age, comorbidities, and medication.

	Endometriosis Cohort			Crude		Adjusted ^†^	
	CAD Events	PY	IR	HR (95% CI)	*p*-Value	HR (95% CI)	*p*-Value
Age, years							
<40	230	84,798	2.71	1.00 (reference)	-	1.00 (reference)	-
40–49	505	51,333	9.84	3.63 (3.10, 4.24)	<0.001	2.96 (2.50, 3.50)	<0.001
50–64	118	7038	16.77	6.19 (4.96, 7.74)	<0.001	3.88 (3.04, 4.95)	<0.001
Comorbidity							
Obesity							
No	831	141,770	5.86	1.00 (reference)	-	1.00 (reference)	-
Yes	22	1399	15.73	2.67 (1.75, 4.08)	<0.001	1.85 (1.20, 2.85)	0.005
CKD							
No	844	142,741	5.91	1.00 (reference)	-	1.00 (reference)	-
Yes	9	428	21.03	3.54 (1.84, 6.83)	<0.001	1.50 (0.77, 2.91)	0.234
Hypertension							
No	632	133,732	4.73	1.00 (reference)	-	1.00 (reference)	-
Yes	221	9437	23.42	4.95 (4.24, 5.77)	<0.001	2.57 (2.15, 3.07)	<0.001
Hyperlipidemia							
No	735	135,336	5.43	1.00 (reference)	-	1.00 (reference)	-
Yes	118	7833	15.06	2.77 (2.28, 3.36)	<0.001	1.26 (1.01, 1.58)	0.038
DM							
No	734	136,463	5.38	1.00 (reference)	-	1.00 (reference)	-
Yes	119	6706	17.74	3.29 (2.71, 4.00)	<0.001	1.95 (1.55, 2.44)	<0.001
Hysterectomy							
No	540	105,475	5.12	1.00 (reference)	-	1.00 (reference)	-
Yes	313	37,694	8.3	1.63 (1.42, 1.87)	<0.001	0.97 (0.84, 1.13)	0.699
Oophorectomy							
No	662	104,720	6.32	1.00 (reference)	-	1.00 (reference)	-
Yes	191	38,449	4.97	0.79 (0.67, 0.92)	0.004	0.91 (0.77, 1.07)	0.24
Medication							
Hormone (estradiol, premarin)							
No	342	56,806	6.02	1.00 (reference)	-	1.00 (reference)	-
Yes	511	86,363	5.92	0.98 (0.86, 1.13)	0.824	0.86 (0.75, 0.99)	0.032
Statin							
No	704	127,306	5.53	1.00 (reference)	-	1.00 (reference)	-
Yes	149	15,863	9.39	1.70 (1.43, 2.03)	<0.001	0.70 (0.57, 0.87)	<0.001
Aspirin							
No	379	75,191	5.04	1.00 (reference)	-	1.00 (reference)	-
Yes	474	67,978	6.97	1.38 (1.21, 1.58)	<0.001	1.19 (1.03, 1.37)	0.016
Antihypertensive							
No	157	54,941	2.86	1.00 (reference)	-	1.00 (reference)	-
Yes	696	88,228	7.89	2.77 (2.33, 3.29)	<0.001	1.88 (1.56, 2.26)	<0.001
Antihyperglycemic							
No	755	131,763	5.73	1.00 (reference)	-	1.00 (reference)	-
Yes	98	11,406	8.59	1.50 (1.22, 1.85)	<0.001	0.68 (0.52, 0.89)	0.005
Insulin							
No	813	140,141	5.8	1.00 (reference)	-	1.00 (reference)	-
Yes	40	3028	13.21	2.27 (1.66, 3.12)	<0.001	1.13 (0.76, 1.66)	0.546

IR: incidence rates per 1000 person-years; HR: hazard ratio; CI: confidence interval; †: Model was adjusted for age and comorbidities listed in Table 1. CAD: coronary artery disease; CKD: coronary kidney disease; DM: diabetes mellitus.

## Data Availability

All relevant data were showed in the manuscript.

## References

[1] Taylor H.S., Kotlyar A.M., Flores V.A. (2021). Endometriosis Is a Chronic Systemic Disease: Clinical Challenges and Novel Innovations. Lancet.

[2] Yamamoto A., Johnstone E.B., Bloom M.S., Huddleston H.G., Fujimoto V.Y. (2017). A Higher Prevalence of Endometriosis among Asian Women Does Not Contribute to Poorer IVF Outcomes. J. Assist. Reprod. Genet..

[3] Yen C.-F., Kim M.-R., Lee C.-L. (2019). Epidemiologic Factors Associated with Endometriosis in East Asia. Gynecol Minim. Invasive Ther..

[4] Andrews W.C., Buttram V.C., Weed J.C., Hammond C.B., Thomas H.H., Behrman S.J., Carmichael E., Cohen M.R., Dmowski P., Eward R.D. (1985). Revised American Fertility Society Classification of Endometriosis: 1985. Fertil. Steril..

[5] Santanam N., Song M., Rong R., Murphy A.A., Parthasarathy S. (2002). Atherosclerosis, Oxidation and Endometriosis. Free Radic. Res..

[6] Kvaskoff M., Mu F., Terry K.L., Harris H.R., Poole E.M., Farland L., Missmer S.A. (2015). Endometriosis: A High-Risk Population for Major Chronic Diseases?. Hum. Reprod. Update.

[7] Mu F., Rich-Edwards J., Rimm E.B., Spiegelman D., Missmer S.A. (2016). Endometriosis and Risk of Coronary Heart Disease. Circ. Cardiovasc. Qual. Outcomes.

[8] Mu F., Rich-Edwards J., Rimm E.B., Spiegelman D., Forman J.P., Missmer S.A. (2017). Association Between Endometriosis and Hypercholesterolemia or Hypertension. Hypertension.

[9] Tiniakou E., Costenbader K.H., Kriegel M.A. (2013). Sex-Specific Environmental Influences on the Development of Autoimmune Diseases. Clin. Immunol..

[10] Kim H.S., Kim T.H., Chung H.H., Song Y.S. (2014). Risk and Prognosis of Ovarian Cancer in Women with Endometriosis: A Meta-Analysis. Br. J. Cancer.

[11] Cirillo M., Coccia M.E., Petraglia F., Fatini C. (2021). Role of Endometriosis in Defining Cardiovascular Risk: A Gender Medicine Approach for Women’s Health. Hum. Fertil..

[12] Agic A., Xu H., Finas D., Banz C., Diedrich K., Hornung D. (2006). Is Endometriosis Associated with Systemic Subclinical Inflammation?. Gynecol. Obstet. Investig..

[13] Scutiero G., Iannone P., Bernardi G., Bonaccorsi G., Spadaro S., Volta C.A., Greco P., Nappi L. (2017). Oxidative Stress and Endometriosis: A Systematic Review of the Literature. Oxid. Med. Cell. Longev..

[14] Melo A.S., Rosa-e-Silva J.C., Rosa-e-Silva A.C.J.D.S., Poli-Neto O.B., Ferriani R.A., Vieira C.S. (2010). Unfavorable Lipid Profile in Women with Endometriosis. Fertil. Steril..

[15] Pathak L.A., Shirodkar S., Ruparelia R., Rajebahadur J. (2017). Coronary Artery Disease in Women. Indian Heart J..

[16] Aggarwal A., Srivastava S., Velmurugan M. (2016). Newer Perspectives of Coronary Artery Disease in Young. World J. Cardiol..

[17] Wilmot K.A., O’Flaherty M., Capewell S., Ford E.S., Vaccarino V. (2015). Coronary Heart Disease Mortality Declines in the United States From 1979 Through 2011: Evidence for Stagnation in Young Adults, Especially Women. Circulation.

[18] Harvey R.E., Coffman K.E., Miller V.M. (2015). Women-Specific Factors to Consider in Risk, Diagnosis and Treatment of Cardiovascular Disease. Womens. Health.

[19] Sachdev M., Sun J.L., Tsiatis A.A., Nelson C.L., Mark D.B., Jollis J.G. (2004). The Prognostic Importance of Comorbidity for Mortality in Patients with Stable Coronary Artery Disease. J. Am. Coll. Cardiol..

[20] Jankowski J., Floege J., Fliser D., Böhm M., Marx N. (2021). Cardiovascular Disease in Chronic Kidney Disease: Pathophysiological Insights and Therapeutic Options. Circulation.

[21] Hajar R. (2017). Risk Factors for Coronary Artery Disease: Historical Perspectives. Heart Views.

[22] Ambrose J.A., Singh M. (2015). Pathophysiology of Coronary Artery Disease Leading to Acute Coronary Syndromes. F1000Prime Rep..

[23] Rea I.M., Gibson D.S., McGilligan V., McNerlan S.E., Alexander H.D., Ross O.A. (2018). Age and Age-Related Diseases: Role of Inflammation Triggers and Cytokines. Front. Immunol..

[24] Giudice L.C., Kao L.C. (2004). Endometriosis. Lancet.

[25] Ramji D.P., Davies T.S. (2015). Cytokines in Atherosclerosis: Key Players in All Stages of Disease and Promising Therapeutic Targets. Cytokine Growth Factor Rev..

[26] Tousoulis D., Oikonomou E., Economou E.K., Crea F., Kaski J.C. (2016). Inflammatory Cytokines in Atherosclerosis: Current Therapeutic Approaches. Eur. Heart J..

[27] Fatkhullina A.R., Peshkova I.O., Koltsova E.K. (2016). The Role of Cytokines in the Development of Atherosclerosis. Biochemistry.

[28] Hogg C., Horne A.W., Greaves E. (2020). Endometriosis-Associated Macrophages: Origin, Phenotype, and Function. Front. Endocrinol..

[29] Tariverdian N., Siedentopf F., Rücke M., Blois S.M., Klapp B.F., Kentenich H., Arck P.C. (2009). Intraperitoneal Immune Cell Status in Infertile Women with and without Endometriosis. J. Reprod. Immunol..

[30] Weisheng B., Nezhat C.H., Huang G.F., Mao Y.-Q., Sidell N., Huang R.-P. (2019). Discovering Endometriosis Biomarkers with Multiplex Cytokine Arrays. Clin. Proteom..

[31] Santoro L., D’Onofrio F., Flore R., Gasbarrini A., Santoliquido A. (2015). Endometriosis and Atherosclerosis: What We Already Know and What We Have yet to Discover. Am. J. Obstet. Gynecol..

[32] Santoro L., D’Onofrio F., Campo S., Ferraro P.M., Flex A., Angelini F., Forni F., Nicolardi E., Campo V., Mascilini F. (2014). Regression of Endothelial Dysfunction in Patients with Endometriosis after Surgical Treatment: A 2-Year Follow-up Study. Hum. Reprod..

[33] Tani A., Yamamoto S., Maegawa M., Kunimi K., Matsui S., Keyama K., Kato T., Uemura H., Kuwahara A., Matsuzaki T. (2015). Arterial Stiffness Is Increased in Young Women with Endometriosis. J. Obstet. Gynaecol..

[34] Ittaman S.V., VanWormer J.J., Rezkalla S.H. (2014). The Role of Aspirin in the Prevention of Cardiovascular Disease. Clin. Med. Res..

[35] De Gaetano G., Collaborative Group of the Primary Prevention Project (2001). Low-Dose Aspirin and Vitamin E in People at Cardiovascular Risk: A Randomised Trial in General Practice. Collaborative Group of the Primary Prevention Project. Lancet.

[36] Hansson L., Zanchetti A., Carruthers S.G., Dahlöf B., Elmfeldt D., Julius S., Ménard J., Rahn K.H., Wedel H., Westerling S. (1998). Effects of Intensive Blood-Pressure Lowering and Low-Dose Aspirin in Patients with Hypertension: Principal Results of the Hypertension Optimal Treatment (HOT) Randomised Trial. Lancet.

[37] The Medical Research Council’s General Practice Research Framework (1998). Thrombosis Prevention Trial: Randomised Trial of Low-Intensity Oral Anticoagulation with Warfarin and Low-Dose Aspirin in the Primary Prevention of Ischaemic Heart Disease in Men at Increased Risk. Lancet.

[38] (1989). Steering Committee of the Physicians’ Health Study Research Group Final Report on the Aspirin Component of the Ongoing Physicians’ Health Study. N. Engl. J. Med..

[39] Ridker P.M., Cook N.R., Lee I.-M., Gordon D., Gaziano J.M., Manson J.E., Hennekens C.H., Buring J.E. (2005). A Randomized Trial of Low-Dose Aspirin in the Primary Prevention of Cardiovascular Disease in Women. N. Engl. J. Med..

[40] Li M., Wang X., Li X., Chen H., Hu Y., Zhang X., Tang X., Miao Y., Tian G., Shang H. (2019). Statins for the Primary Prevention of Coronary Heart Disease. Biomed. Res. Int..

[41] Vercellini P., Eskenazi B., Consonni D., Somigliana E., Parazzini F., Abbiati A., Fedele L. (2011). Oral Contraceptives and Risk of Endometriosis: A Systematic Review and Meta-Analysis. Hum. Reprod. Update.

[42] Arnold S.V., Bhatt D.L., Barsness G.W., Beatty A.L., Deedwania P.C., Inzucchi S.E., Kosiborod M., Leiter L.A., Lipska K.J., Newman J.D. (2020). Clinical Management of Stable Coronary Artery Disease in Patients With Type 2 Diabetes Mellitus: A Scientific Statement From the American Heart Association. Circulation.

[43] Chiang H.-J., Lan K.-C., Yang Y.-H., Chiang J.Y., Kung F.-T., Huang F.-J., Lin Y.-J., Su Y.-T., Sung P.-H. (2021). Risk of Major Adverse Cardiovascular and Cerebrovascular Events in Taiwanese Women with Endometriosis. J. Formos. Med. Assoc..

[44] Waller K.G., Shaw R.W. (1998). Risk Factors for Endometriosis. Med. Princ. Pract..

[45] Parazzini F., Vercellini P., Pelucchi C. (2012). Endometriosis: Epidemiology, and Etiological Factors. Endometriosis.

[46] Treloar S.A., Bell T.A., Nagle C.M., Purdie D.M., Green A.C. (2010). Early Menstrual Characteristics Associated with Subsequent Diagnosis of Endometriosis. Am. J. Obstet. Gynecol..

